# Emerging Risk for Human T-Cell Leukemia Virus Type 1 Transmission with HIV-Positive Breastfeeding Support 

**DOI:** 10.3201/eid3207.251525

**Published:** 2026-07

**Authors:** Agnes Meybeck, Nathalie Viget, Emmanuelle Aïssi, Roxane Vanspranghels, Yasmine Clermont-Hama, Marion Lagrée, Enagnon Kazali Alidjinou, Olivier Robineau

**Affiliations:** Centre Hospitalier de Tourcoing, Tourcoing, France (A. Meybeck, N. Viget, E. Aïssi, Y. Clermont-Hama, O. Robineau); Centre Hospitalier Universitaire de Lille, Lille, France (R. Vanspranghels, M. Lagrée, E.K. Alidjinou); Université de Lille, Lille (E.K. Alidjinou, O. Robineau).

**Keywords:** human T-cell lymphotropic virus type 1, HTLV-1, mother-to-child transmission, mother-to-child transmission, prevention, HIV/AIDS, viruses, viral infections

## Abstract

Human T-cell leukemia virus 1 (HTLV-1) is a neglected retrovirus affecting 5–10 million persons worldwide. Most infections are asymptomatic, but HTLV-1 can cause adult T-cell leukemia or lymphoma and HTLV-1–associated myelopathy. Although mother-to-child transmission through breastfeeding is preventable, few countries have policies that include antenatal screening. The World Health Organization recommends integrating HTLV-1 into HIV and sexually transmitted infection strategies. HIV guidelines in high-income countries increasingly support breastfeeding under controlled conditions, creating increased risk for unrecognized HTLV-1 transmission. We reviewed existing policies for HTLV-1 mother-to-child transmission and considered integration of HTLV prevention into HIV response. We discovered inconsistent guidance across HIV, pediatric, and obstetric fields, leading to conflicting counseling of expectant mothers. Integration of HTLV-1 prevention into HIV guidelines and harmonization with maternal and child health policies is essential for awareness among providers. Prevention through screening and avoiding breastfeeding remains the cornerstone of HTLV-1 control.

Human T-cell leukemia virus 1 (HTLV-1) remains a neglected retrovirus, despite its potentially severe consequences: adult T-cell leukemia or lymphoma and HTLV-1–associated myelopathy. In addition to those 2 severe diseases, >14 clinical conditions have been shown to be associated with HTLV-1, including infective dermatitis, uveitis, lung disease, tuberculosis, and severe strongyloidiasis. Persons with HTLV-1 experienced higher risk of death (*1*). Transmission occurs through breastfeeding, sexual contact, needle sharing, and blood products (*2*). Most persons infected with HTLV-1 remain asymptomatic. The risk for adult T-cell leukemia or lymphoma, the most severe disease caused by HTLV-1, is estimated to be up to 20% after early life infection (*3*). HTLV-1 has no current effective treatment; therefore, emphasis should be placed on detection and prevention. We reviewed knowledge about HTLV-1 mother-to-child transmission (MTCT) and existing policies for HTLV-1 prevention and consider why and how the HIV management field should play a role in the prevention of HTLV-1 MTCT.

## Epidemiology of HTLV-1 Infection and Risk for MTCT

The total estimate of people living with HTLV-1 infection ranged from 5 to 10 million in 2012, but that number was probably underestimated (*2*). In collaboration with member states and partners, the World Health Organization (WHO) works to develop guidance on HTLV-1 surveillance methods, including those used to determine prevalence. Distribution of HTLV-1 infection prevalence varies widely throughout the world. High-prevalence countries are defined by a prevalence in the general population of >1%. Regions identified as high endemicity areas include southern Japan, the Caribbean, areas of South America and tropical Africa, and foci in the Middle East, Australia, and Melanesia (*4*). In Europe, prevalence is low except in Romania and possibly Moldova (*4*,*5*).

HTLV-1 is mainly transmitted sexually (more often male-to-female) or vertically by breastfeeding. Approximately 20% of breastfed infants of HTLV-1 infected mothers acquire HTLV-1, compared with <5% if breastfeeding is avoided (*2*,*6*).

## Existing Policies and Guidelines for Prevention of HTLV-1 MTCT

Recently, WHO has recognized HTLV-1 as a global concern and listed prevention of HTLV-1 MTCT as a goal for 2030 (*7*). WHO has undertaken consultations with member states and partners and published a 2021 technical report that summarized existing policies and guidelines for preventing HTLV-1 transmission and treating those with HTLV-1–associated diseases (*8*). According to the report, Japan has implemented a nationwide program for preventing HTLV-1 MTCT, including universal antenatal screening and counseling for breastfeeding avoidance if positive (*9*). Some territories in the Caribbean also screen pregnant women for HTLV-1 (e.g., Grenada, Saint Lucia), and the Organisation of Eastern Caribbean States recommends screening pregnant women and exclusive formula feeding (*10*). Among high-prevalence countries, Seychelles also screens all pregnant women for HTLV-1 and recommends avoiding breastfeeding in case of positivity. The Pan American Health Organization (PAHO) and its member states established targets and goals for the elimination of HTLV vertical transmission through public health programs. The PAHO goal is to achieve a <5% of vertical transmission rate through an expected HTLV screening coverage of 95% of pregnant women and >90% of babies exposed to possible vertical transmission and intervention implementation of >90% to prevent transmission (*11*). 

Among low-prevalence countries, France recommends screening of breast milk donors and pregnant women from endemic regions, such as the Caribbean, Africa, Japan, and Southeast Asia (*12*). Despite those recommendations, implementation of HTLV-1 antenatal screening is low in France except in overseas territories in South America (French Guiana) and the Caribbean (Martinique and Guadeloupe) (*13*). In the United Kingdom, screening of all breast milk donors is mandatory (*14*). However, the UK National Screening Committee advised against HTLV-1 universal antenatal screening and is currently assessing targeted antenatal screening for pregnant women who are at high risk for living with HTLV-1 (*15*). Testing for HTLV-1 is already recommended in women from high endemic regions in the UK health migrants guide (*16*). Experts from Spain advocate for expanding HTLV-1 screening in Europe (*17*).

## Epidemiologic Similarities Between HIV and HTLV-1

WHO suggested integrating HTLV-1 control measures into existing HIV and sexually transmitted infection interventions (*7*). A first step of this implementation could be HTLV-1 antenatal screening in women living with HIV, in agreement with guidelines that recommend HTLV-1 testing in high-risk pregnant women. HTLV-1 and HIV share the same transmission routes and similar high prevalence regions. In a study conducted in Brazil in several epidemiologically relevant groups, the prevalence of HTLV-1 infection was higher among HIV-positive persons (*18*). In Gabon, screening for HTLV-1 infection in those living with HIV revealed a high rate of co-infections, reaching 7% of the population (*19*). Co-infections were far more prevalent in women. In HTLV-1 low prevalence countries, HTLV-1 infection might be higher in women living with HIV. The last report from the European Centre for Disease Prevention and Control regarding HIV/AIDS surveillance revealed an increased number of HIV diagnoses in the European Union and European economic area and countries to the west of the region in 2023 compared with 2022. The increase is primarily because of a rise in persons diagnosed with HIV originating from sub-Saharan Africa and continued high rates among those originating from central and eastern Europe, Latin America, and the Caribbean (*20*). Several studies conducted in Europe suggest a higher prevalence of HTLV-1 infection in migrants, especially in women (*21*). In France, similar to the United Kingdom, HTLV-1 testing is included in the health screen of newly arrived migrant women of reproductive age (*16*,*22*). The European Centre for Disease Prevention and Control, however, did not mention HTLV-1 infection in its guidelines on screening and vaccination for infectious diseases in newly arrived migrants within the European Union and European economic area (*23*).

## HIV and Breastfeeding, the Evolving Context

Current WHO guidelines recommend lifelong antiretroviral treatment for pregnant and breastfeeding women living with HIV. Mothers living with HIV who are on antiretroviral therapy should exclusively breastfeed their infants for the first 6 months, introducing appropriate complementary foods thereafter, and continue breastfeeding for >12 months (*24*). In low- and middle-income countries, the decision regarding whether mothers living with HIV should breastfeed is made on the basis of balancing the risk for HIV transmission through breastfeeding with the increased risk for illness and death in the absence of a safe diet without breast milk. Most Latin American countries recommend mothers living with HIV to avoid breastfeeding and provide infant formula (*25*). Exclusive breastfeeding for the first 6 months is an alternative guideline when formula feeding is not acceptable, feasible, affordable, sustainable, and safe (*25*). In Guatemala, mothers are given the option to choose breastfeeding or formula feeding after being fully informed about the risks and benefits of different feeding methods. Argentina and the Caribbean nation of Trinidad and Tobago have recently updated their breastfeeding HIV guidelines to state that mothers living with HIV who choose to breastfeed are closely monitored and should adhere strictly to antiretroviral treatment (*25*). Similarly, recent changes in high-income countries HIV guidelines have marked a shift in favor of breastfeeding under controlled conditions. For decades, formula feeding was the standard, but the accumulated evidence has demonstrated that the risk for HIV transmission is negligible when undetectable viremia is maintained during pregnancy. Because of this negligible risk, countries such as the United Kingdom and Switzerland now recommend or enable breastfeeding with appropriate monitoring (*26*,*27*). A survey conducted in 2022 by the European AIDS Clinical Society revealed that 23 of 25 countries in Europe had specifically mentioned breastfeeding in their HIV and pregnancy guidelines (*28*). Among those countries, 11 offered breastfeeding as an option under certain conditions. Since publication of the survey, France has also begun enabling breastfeeding in women living with HIV under strict virologic and clinical criteria (*29*). The last update of European AIDS Clinical Society guidelines, however, generally discouraged breastfeeding, stating it should only be considered if maternal viral load is undetectable and with close follow-up (*30*). Similarly, the US Department of Health and Human Services, the Canadian Paediatric and Perinatal HIV/AIDS Research Group, and the Australasian Society for HIV, Viral Hepatitis and Sexual Health Medicine continue to support exclusive formula feeding as the preferred method for infants born to women living with HIV; although they have included in their guidelines breastfeeding options for women on antiretroviral therapy with sustained undetectable viral load (*31*–*33*).

## Prevention of HTLV-1 MTCT in Guidelines

This evolution in practice regarding HIV and breastfeeding requires maintaining attention to other vertically transmitted viruses, particularly HTLV-1 (*34*). Indeed, there is a potential overlap of women living with HIV in high-income countries and those at high risk for having HTLV-1 infection. WHO’s guidance is clear: HIV-positive women on antiretroviral treatment with suppressed viral load should breastfeed for >12 months in low- and middle-income countries (*24*). WHO does not have guidance yet regarding HTLV-1 MTCT. In WHO’s technical report summarizing existing policies for preventing HTLV-1 transmission, breastfeeding appeared to be contraindicated for HTLV-1–infected mothers (*8*). Similarly, in the technical note of PAHO on good practices, the preferred recommendation for HTLV-1–infected mothers is exclusive formula feeding, short-term breastfeeding being an alternative in settings where formula feeding is not acceptable, feasible, affordable, sustainable, and safe (ideally <3 months) (*11*). Because HIV care increasingly includes breastfeeding in high-income countries, the risk of unrecognized HTLV-1 transmission in migrant or co-infected populations is real (*18*,*19*,*21*). Pregnant women living with HIV, especially those from HTLV-1 endemic regions, should be offered HTLV-1 screening as part of routine antenatal care. Early diagnosis enables informed counseling and infant feeding alternatives.

HTLV-1 prevention messages remain fragmented across disciplines (*22*,*35*). Pediatric and gyneco-obstetric guidelines might recommend screening of at-risk women or infants and contraindicate breastfeeding in infants born to mothers infected with HTLV-1, yet HIV-specific guidelines often fail to mention HTLV-1 altogether (*29*,*30*,*35*–*45*). High-income countries should take advantage of low- and middle-income countries’ experience. In Brazil, for example, HTLV-1 testing is recommended for all persons living with HIV, at baseline visit, in the HIV clinical protocol and therapeutic guidelines (*46*). In the Organisation of Eastern Caribbean States guidelines for sexually transmitted infections, HTLV testing is recommended for anyone living with HIV (*47*).

We illustrate the divergence between HIV guidelines, which increasingly support breastfeeding, and national breastfeeding guidelines, and often contraindicate it for HTLV-1 (Appendix Table). Only Japan integrated HTLV-1 into both HIV and maternal–child health policies. In addition, HIV guidelines almost never mention HTLV-1, whereas obstetric and pediatric guidance might list it as a formal contraindication. In 2024, the PAHO included HTLV-1 in the mother-to-child HIV, syphilis, hepatitis B and C, and Chagas elimination protocol (*11*).

Integration of HTLV-1 information into HIV guidelines is essential but should also be accompanied by dissemination through obstetric, midwife, pediatric, infectious disease, and migrant healthcare pathways to ensure visibility and implementation (*7*,*8*). Implementation of counseling regarding HTLV-1 could be easier in the context of HIV infection, because breastfeeding counseling on transmissible infections is already routinely done (*48*). Implementation could also enable specific research, including evaluating the effect of certain antiretroviral treatments and their role in elimination of HTLV-1 MTCT (*49*,*50*).

In conclusion, HTLV-1 MTCT is preventable but neglected. Because HIV guidelines increasingly support breastfeeding, not including HTLV-1 represents a missed opportunity and a public health risk. Women living with HIV, particularly from HTLV-1–endemic regions, should be systematically offered HTLV-1 screening as part of antenatal care. To be effective, prevention strategies should not remain isolated. Articulation and harmonization between HIV, obstetrics, pediatrics, and public health guidelines are urgently needed to ensure consistent counseling, avoid contradictory messages, and to raise awareness among all healthcare providers. Leveraging HIV MTCT programs offers the most practical pathway to reduce HTLV-1 transmission. The HIV field has the expertise and infrastructure to lead the way. Failure to integrate HTLV-1 prevention can result in a silent epidemic of preventable infections.

Appendix Additional information about emerging risk for human T-cell leukemia virus type 1 transmission with HIV-positive breastfeeding support.
