# Supplementary material for: Emerging Risk for Human T-Cell Leukemia Virus Type 1 Transmission with HIV-Positive Breastfeeding Support
Source: Emerg Infect Dis. 2026 Jul;32(7):1052–7. doi: 10.3201/eid3207.251525 (PMC13322437; doi:10.3201/eid3207.251525)
Supplement: Appendix — Additional information about emerging risk for human T-cell leukemia virus type 1 transmission with HIV-positive breastfeeding support. [file 25-1525-Techapp-s1.pdf]

Article DOI: https://doi.org/10.3201/eid3207.251525

EID cannot ensure accessibility for supplementary materials supplied by authors. Readers who have difficulty accessing supplementary content should contact the authors for assistance.

Emerging Risk for Human T-Cell Leukemia Virus Type 1 Transmission with HIV-Positive Breastfeeding Support

Appendix.

Appendix Table. Breastfeeding guidelines of selected high-income countries: HTLV-1 consideration in general population and among HIV-infected women. Comparaison with WHO and PAHO guidelines*

Region /Country	HTLV-1 prevalence in pregnant women	Screening guidelines in pregnancy	Breastfeeding guidelines in general population	HTLV-1 consideration	Breastfeeding guidelines in HIV-infected women and HTLV-1 consideration
WHO	Not applicable	Under development (*8*)	Exclusive breastfeeding for the first 6 mo; introduction of appropriate complementary food thereafter; continuation of breastfeeding for at least 12 mo (up to 24 mo)	No formal guidance (*8*)	Exclusive breastfeeding for the first 6 mo; introduction of appropriate complementary food thereafter; wean when safe replacements are available (*24*)<br>No specific consideration for HTLV-1
PAHO	Not applicable	HTLV-1 antenatal screening (*11*)	Exclusive breastfeeding for the first 6 mo; introduction of appropriate complementary food thereafter; continuation of breastfeeding for at least 12 mo (up to 24 mo).	Exclusive formula feeding (preferred); Short-term breastfeeding in context where formula feeding is not AFASS (*11*)	Avoid breastfeeding ; Exclusive breastfeeding for the first 6 mo in context where formula feeding is not AFASS ; introduction of appropriate complementary food thereafter (*25*)<br>HTLV-1 is included in the EMTCT Plus protocol (*11*)
Australasia	No data ; Prevalence of 6% in children in Central Australian Aboriginal communities (2)	All aboriginal pregnant women should be offered information about HTLV-1, including the availability of testing (47)	Exclusive breastfeeding until around 6 mo of age when solid foods are introduced (*37*)	HTLV-1 not mentioned in general population guidelines (*37*). Specific guidelines for aboriginal primary health care settings: all pregnant women with HTLV-1 should be provided with lactation and feeding advice and resources to provide formula feeding to their baby if they choose formula feeding (47)	Breastfeeding in case of optimal scenario: adherence to ART, ongoing clinical care, HIV pVL<50 copies/ml throughout the pregnancy and breastfeeding ; Otherwise formula feeding<br>HTLV-1 not mentioned (*33*)
Canada	No data	No guidance (8)	Exclusive breastfeeding for the first six months (*38*)	Exclusive replacement feeding recommended in women living with HTLV-1 (*38*)	Exclusive replacement feeding recommanded ; Individualized and multi-disciplinary approach to breastfeeding under certain circumstances, including strict adherence to ART;

Region /Country	HTLV-1 prevalence in pregnant women	Screening guidelines in pregnancy	Breastfeeding guidelines in general population	HTLV-1 consideration	Breastfeeding guidelines in HIV-infected women and HTLV-1 consideration
Europe	No data	No guidance (8)	Support exclusive breastfeeding for the first six months (*37*)	Breastfeeding is contraindicated in infants born to mothers affected by HTLV-1 and 2 infections (*39*)	HTLV-1 not mentioned (*32*) Generally discourage breastfeeding; may support it if maternal pVL undetectable and with close follow-up HTLV-1 not mentioned (30)
France	In main land: 0.1% in Paris ; In overseas territories: Guadeloupe 3%, Martinique 2.4% French Guiana 4.2% (*2*)	screening of breast-milk donors and pregnant women from endemic regions (*8*)	Exclusive breastfeeding on demand for 4 to 6 mo is recommended (*40*)	HTLV-1 infection contraindicates breastfeeding (*40*)	Formula feeding; Breastfeeding possible if adherence to ART, ongoing clinical care, HIV pVL<50 copies/ml throughout the pregnancy and breastfeeding, and reinforced follow-up throughout the duration of breastfeeding HTLV-1 not mentioned (*29*)
Japan	National prevalence: 0.14%, prevalence in Kyushu: 0.6% (2)	Nationwide policy since 2011: universal antenatal screening (*8*,*9*)	Exclusive breastfeeding recommended until 6 mo	HTLV-1 infection contraindicates breastfeeding (*8*)	Infant formula feeding in infants born to mothers affected by HIV or HTLV-1 (*41*)
Switzerland	No data	No guidance	Breatfeeding for at least 4 mo is recommended (*42*)	HTLV-1 not mentioned (*42*)	Supported breastfeeding permitted with undetectable pVL and follow-up HTLV not included in current national HIV breastfeeding guidance (*27*)
USA	No data	No guidance	exclusive breastfeeding for 6 mo followed by continued breastfeeding with complementary foods for at least 2 y	HTLV listed as a maternal contraindication to breastfeeding (*43*)	Breastfeeding only if maternal pVL <50 copies/mL on ART; otherwise use replacement feeding HTLV is not mentioned in HIV feeding policies (*31*)
UK	Prevalence of 0.3% (2)	Breast-milk donation screening (*8*)	Exclusive breastfeeding is recommended for the first six months (*44*)	Migrants health guidance on women's health refers to avoidance of breastfeeding for women living with HTLV (*16*)	Formula feeding recommended supported breastfeeding allowed under pVL suppression and monthly monitoring HTLV-1 not mentioned (*26*)

*AFASS: acceptable, feasible, affordable, sustainable, and safe ; ART: antiretroviral therapy; pVL: plasma viral load.
